# Involvement of Corticotropin-Releasing Factor and Receptors in Immune Cells in Irritable Bowel Syndrome

**DOI:** 10.3389/fendo.2018.00021

**Published:** 2018-02-12

**Authors:** Mahanand Chatoo, Yi Li, Zhiqiang Ma, John Coote, Jizeng Du, Xuequn Chen

**Affiliations:** ^1^Division of Neurobiology and Physiology, Department of Basic Medical Sciences, School of Medicine, Zhejiang University, Hangzhou, China; ^2^School of Clinical and Experimental Medicine, University of Birmingham, Birmingham, United Kingdom; ^3^Key Laboratory of Medical Neurobiology of the Ministry of Health, Institute of Neuroscience, School of Medicine, Zhejiang University, Hangzhou, China; ^4^Key Laboratory of Medical Neurobiology of Zhejiang Province, Institute of Neuroscience, School of Medicine, Zhejiang University, Hangzhou, China

**Keywords:** corticotropin-releasing factor, CRFR1, CRFR2, inflammation, irritable bowel syndrome

## Abstract

Irritable bowel syndrome (IBS) is a common functional gastrointestinal disorder defined by ROME IV criteria as pain in the lower abdominal region, which is associated with altered bowel habit or defecation. The underlying mechanism of IBS is not completely understood. IBS seems to be a product of interactions between various factors with genetics, dietary/intestinal microbiota, low-grade inflammation, and stress playing a key role in the pathogenesis of this disease. The crosstalk between the immune system and stress in IBS mechanism is increasingly recognized. Corticotropin-releasing factor (CRF), a major mediator in the stress response, is involved in altered function in GI, including inflammatory processes, colonic transit time, contractile activity, defecation pattern, pain threshold, mucosal secretory function, and barrier functions. This mini review focuses on the recently establish local GI-CRF system, its involvement in modulating the immune response in IBS, and summarizes current IBS animal models and mapping of CRF, CRFR1, and CRFR2 expression in colon tissues. CRF and receptors might be a key molecule involving the immune and movement function *via* brain–gut axis in IBS.

## Introduction

Functional gastrointestinal disorders (FGIDs) are a group of idiopathic disorders which affect different parts of the gastrointestinal (GI) tract. They are classified by GI symptoms related to any combination of the following: motility disturbance, visceral hypersensitivity, altered mucosal and immune function, altered gut microbiota, and altered central nervous system (CNS) processing. The FGIDs are classified into six major domains for adults including irritable bowel syndrome (IBS) which is in FGIDs-C. IBS is defined by ROME IV criteria as pain in the lower abdominal region, which is associated with altered bowel habit or defecation (1, 2). Patients are diagnosed according to symptom-based criteria and the majority of the time they report recurring pain in the lower abdomen, accompanied by altered stool form or frequency (2, 3). These symptoms occur without any known structural abnormalities (3, 4). IBS is further subcategorized as diarrhea predominant (IBS-D), constipation predominant (IBS-C), alternating (IBS-A), or unspecified (IBS-U) (1, 2). The global prevalence of IBS is 11.2% making it an important clinical entity, but the underlying mechanism is not fully understood (5).

## Possible Causes of IBS

Irritable bowel syndrome is a multifactorial disorder with diet/luminal microbiota, low-grade inflammation, stress, and genetics. IBS patients report a worsening of symptoms after eating specific food such as diary product, hot spices, and wheat product. Recent area of interest relates the effect of altered intestinal bacterial flora namely increased *Firmicutes* and reduced *Bacteroidetes, Lactobacillus, Bifdobacteriumsp*, and *Bifidobacter* to the onset IBS (1, 6). There is evidence that the luminal microbiota affects GI motility by interacting with muscularis macrophage and enteric neurons (7). In a prospective trial, IBS patients receiving *Bifidobacteriumlongum* showed decreased-depression scores, improved quality of life scores, and overall symptoms (8). Investigators have also suggested that small-intestinal bacterial overgrowth might contribute to IBS progression (9).

There is evidence to support the role of mild intestinal inflammation in the etiology of IBS. Researchers have found alteration in inflammatory mediators in the GI tract as well as in the peripheral blood circulation of animal and patient with IBS. Patients reported the onset of postinfectious IBS, a subset of IBS, after contracting viral, bacterial, protozoa, and nematode infections (6, 9). There are experimental models showing that inflammation, even if mild, could lead to long-term changes in GI nerve and smooth muscle function, resulting in dysmotility, hypersensitivity (1, 7, 9, 10).

ROME IV uses a biopsychosocial conceptual model to explain the susceptibility to develop IBS. This means that IBS is the product of interactions between various factors and stress (1). Psychological and physical stresses exacerbate GI symptoms. IBS patients also suffer from psychiatric disorders such as anxiety and depression (9, 11). Animal and human studies have demonstrated that stress stimulates colonic motor function, reflected by decreased-colonic transit time, increased contractile activity, the induction of defecation, and symptoms of diarrhea. There is also evidence to support that stress affects gut-pain threshold, mucosal secretory function, barrier functions, and visceral inflammatory response (1, 4).

Irritable bowel syndrome patients more often have a family history of IBS (1). A search for candidate genes to reinforce the hypothesis that environmental factors play an important role in the pathogenesis of IBS has led to the association of serotonin transporter gene and cholecystokinin A receptor gene with IBS. It has been found that patients with IBS-D have a functional polymorphism in the serotonin transporter gene (6).

## CRF Mechanism in IBS

The hypothalamic-pituitary-adrenal (HPA) axis is crucial in maintaining homeostasis and plays an important role in responses of the endocrine system and behavioral activity to various stresses. Corticotropin-releasing factor (CRF or CRH) plays a pivotal and well-established role in activating the HPA axis under basal and stress conditions (12). There is convergent evidence indicating the presence of CRF, Ucns, CRFR1, and CRFR2 in various peripheral tissues such as GI tract, heart, lungs, spleen, testis and adipose tissue, and CNS. CRF, Ucns, and CRF receptors have been identified in myenteric neuron, sensory nerve, sympathetic nerve, enterochromaffin cell, and immune cells in the intestine of animals and human. This indicates that both central and peripheral CRF systems modulate the body response to stress and modulate syndromes that occurs in IBS (11–14). Early weaning stress in pigs (15–21 days) causes impaired intestinal mucosal function. A decreased-CRF protein, an increased-CRFR2 protein, and no change in CRFR1 protein was detected in jejunum of late weaned pigs. Blocking both CRFR1 and CRFR2 improved disturbances in barrier function, whereas blocking CRFR2 leads to an enhanced barrier dysfunction, showing that dysfunction and hypersecretion is mediated by CRFR1 (15). Central administration of CRF induced colonic hypersensitivity in low-anxiety rats (Fischer 344); this effect was inhibited by pretreatment with CRFR1 antagonist (16). Water-avoidance stress and injection of CRF increased fecal pellet output which is inhibited by CRFR1/R2 antagonist and CRFR1 antagonist CP-154,526 (17). Therefore, CRF *via* its CRF receptors affects smooth muscle contractility, mucosal permeability, mucosal transport, and visceral pain sensitivity, indicating possible correlation with colonic manifestations of IBS (4, 11, 13). To study the mechanism of IBS, many animal models have been developed. Various approaches using chemical, mechanical stimulation, and physiological/psychological stress such as drugs, colorectal distention, restraint stress, maternal separation, water-avoidance stress, electric foot shock, and cold water stomach irrigation (18–27) have been used to study IBS pathogenesis on animal but no ideal animal model has been created up till now. Table 1 is a summary of the currently used stresses in IBS model to study its different hallmarks.

**Table 1:** Stress-induced irritable bowel syndrome (IBS) animal model in the current literature.

IBS-type phenotype	Stresses	Genetic background	Age/weight	Reference
Increased colonic epithelial secretion Increased fecal pellets Increased numbers of abdominal muscle contraction	Restraint stress for 1 h Overnight illumination for 12 h, 45°C hot environment for 5 min, water deprivation for 24 h, 4°C cold environment for 3 min, tail clamp for 1 min, level vibration (120 /min) for 40 min and food deprivation for 24 h.	Wistar	200 ± 20 g	(21)

Inflammation in the colon Visceral hyperalgia Enterochromaffin cell hyperplasia	Trinitrobenzene sulfonic acid was administered: (a) with different doses (20, 10, 5 mg/0.8 mL per rat); (b) with same dose in different concentrations (20 mg/rat, 25, 50 mg/mL); (c) in different ethanol percentage (25%, 50%); and (d) at depth either 4 cm or 8 cm from anus.	Sprague–Dawley	6 weeks	(18)

Visceral pain Slight damage in mucous epithelium with few glands Few inflammatory cell in mucous layer	Colorectal distention; angioplasty balloon 2.5 mm, 60 mmHg daily between age 8–14 days for 1 min (two times within 1 h); angioplasty balloon 3.5 mm, 60 mmHg daily between age 15–22 days for 1 min (two times within 1 h)	Sprague–Dawley	60 days	(19)

Visceral Hypersensitivity	Maternal separation PND 3 to PND 21 for 3 h daily	Sprague–Dawley	8 weeks	(24)

Abnormal colorectal motility Change in colon microbiota	Maternal separation PND 2 to PND 14 for 3 h daily	Wistar	2 days	(23)

Visceral hypersensitivity Increased colonic permeability Altered motility Changes in colon microbiota	Water avoidance stress for 1 h per day for 10 days	Sprague–Dawley	160 ± 20 g	(25)

Increased defecation Stimulate proximal and distal colonic transit.	Conditioned fear stress; electric foot-shock, 5 s/min for 15 exposures	Sprague-Dawley	5–8 weeks	(26)

Increase in number of fecal pellets Increase in weight per fecal pellets	Restraint stress for 1 h	Charles Foster strain albino rats	5–6 weeks	(22)

1.1 Less water content in feces 1.2 Visceral hypersensitivity 1.3 Delayed small-intestine transit rate 2.1 More water content 2.2 Visceral hypersensitivity 2.3 Increase small-intestine transit rate.	0–4°C, 2mL water stomach irrigation daily for 14 days Restraint stress	Sprague–Dawley	200 ± 20 g	(27)

Visceral hypersensitivity	Heterotypic chronic stress for 9 days;60-min water-avoidance stress, 45-min cold restraint stress at 4°C or 20-min forced swimming stress	Wistar rats	6–10 weeks	(20)

## Involvement of CRF Signaling in Immune Cells in IBS

**Figure 1:**
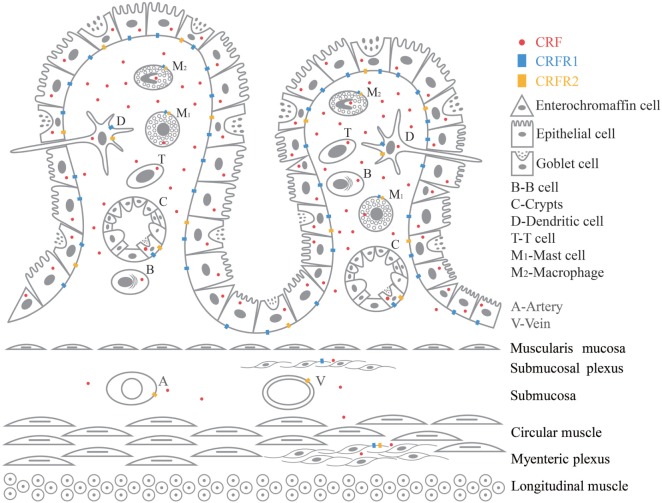
Graphic summary of the expression of CRF, CRFR1, and CRFR2 in GI tissue.

Recent studies demonstrated a novel therapeutic potential of the mechanisms showing complex interactions between immune cells, epithelial cells, smooth muscle, enteric nerves (28), as well as their respective roles in manifestations of clinical symptoms of FGIDs (29). IBS was thought to be a neurological condition as a result of imbalances in brain–gut axis, but there has been growing evidence revealing immunological imbalances in IBS patients with a chronic and a low-grade immune activation (3). CRF promotes inflammation by stimulating release of proinflammatory cytokines TNF-α, IL-1, IL-6, and macrophage inflammatory protein 1α (MIP-1α) from immune cells in GI (11, 13, 30). CRF and CRF receptors in the CNS are also key signals triggering various stress response including the altered visceral response in the stomach, small intestine, and colon (11). There is a close anatomic relationship between neurons from peripheral nervous system (PNS) and ENS and resident immune cells (13, 29). There is growing and compelling evidence proving the correlation between mild inflammatory response and stress in IBS pathogenesis. But so far not much clarification has been given on the role played by the peripheral CRF-receptor signaling in this inflammatory response (11–14). A variety of immune cells are involved in the pathogenesis of IBS. Here, we have selected six immune cells, namely intestinal epithelial cells, macrophages, dendritic cell, mast cell, T cell, and B cell to review. CRF, CRFR1 and CRFR2mRNA, and protein have been found in GI tissues such as blood vessel, B cell/plasma cell (31–35), dendritic cell, enterochromaffin cell (30, 36–41), epithelial cell (39, 40, 42–44), goblet cell (31), intestinal crypt, lamina propria (40, 43), macrophage (32, 35, 39, 42, 45–48), mast cell (49, 50), myenteric plexus (31, 40, 42, 50–52), stem cell of intestinal crypt (31), submucosal plexus (31, 42, 52), and T cell (32–34, 53, 54). Figure 1 is a graphic summary of the expression of CRF, CRFR1, and CRFR2 in GI tissue.

Intestinal epithelial cells protect the body against luminal antigen and pathogens derived from the external environment by producing high amounts of mucus and secreting antimicrobial peptides. Tightly sealed by tight junctions the intestinal epithelial cells allow only small molecules to cross the epithelium *via* the paracellular route (13). They express pattern recognition receptors such as toll-like receptors (TLR) which triggers tissue-specific innate immunity. Furthermore, these cells have the ability to initiate and amplify the immune response by secreting cytokines and chemokines (29). Immunoreactive CRF and CRFmRNA was detected in mucosal epithelial cells in human and rat (39, 40). There was CRFR1 at the base of absorptive surface epithelial cell (42) of sigmoid colon in healthy subjects and CRFR2 was reported in epithelial cell of distal/sigmoid colon biopsy samples (55). Water-avoidance stress or mucosal tissue exposed to CRF causes decreased transepithelial resistance and increased paracellular and transcellular macromolecular, ileal villus epithelium, and follicle-associated epithelium (56) in rat colon. Also in human colon epithelial cell line (HT-29), CRF decreased transepithelial resistance, increased the permeability of horseradish-peroxidase, increased claudin2, and TLR4 expression at the mRNA and protein level (57). Likewise there is higher expression of CRFR1, CRFR2, TLR2, and TLR4 in peripheral blood of IBS patients, supporting that the activation of CRF-TLR may lead to IBS (58). CRF stimulates ion secretion in the distal colon of Wistar–Kyoto rats *in vitro*; this epithelial response was inhibited in tissues pretreated with a non-selective CRF-receptor antagonist, indicating the involvement of CRF in ion secretion (59). CRFR2 antagonist delayed healing, decreased epithelial cell proliferation, increased apoptosis, and proinflammatory cytokine expression in colon of colitis mice; meanwhile, there was an increased proliferation and migration in CRFR2-overexpressing colonic epithelial cells (60).

Macrophages are innate immune cells distributed throughout the GI tract. The function of macrophages is to maintain tissue homeostasis by phagocytizing and clearing invading pathogens and they also act as antigen-presenting cells and secrete a wide range of cytokines (3). Intestinal epithelial cell-secreted mediators and intestinal luminal content have the capability to stimulate mucosal macrophages (29). Recently, a distinct population of macrophages associated with intestinal muscularis externa has been reported which have the ability to modulate colonic peristaltic activity (7, 29). Macrophages-expressed TRLs, and an increased-TLR4 and -TLR5 expression, and a decreased-macrophage number have been found in colonic biopsies of IBS. Macrophage-attracting chemokines and number of CD68^+^ macrophages are decreased in intestinal biopsies of IBS (3). There is evidence indicating that macrophages have the ability to secrete CRF (32, 35) and the CRF participates in immune system function in inflammation (39, 42, 46, 47). RAW264.7 macrophage cells are positive for CRF, CRFR1 and R2 in immunohistochemistry experiment. CRF can enhance the antigen-specific antibody response through the CRFR1 by NF-kappaB (48). CRFR1 immunoreactivity increased in macrophages in the lamina propria (42) in colonic biopsies with ulcerative colitis. Also, CRF, Ucn1, and Ucn2, *via* CRFR1 and CRFR2, increase TNF-α transcription in murine RAW264.7 (13). CRF evoked an enhanced release in proinflammatory cytokines TNF-α and IL-6 from macrophages *in vitro*. CRFR1 antagonist reduced the elevated macrophage-derived TNF-α, IL-1β, and IL-6 in blood after LPS in BALB/c mice (11).

Intestinal dendritic cells shape adaptive immune responses to harmful or infectious intraluminal stimuli through acquisition of luminal antigens and migration to mesenteric lymph nodes to present these antigens to naive T cells. An increased number of intestinal lamina propria dendritic cells, decreased endocytic ability, and enhanced abilities to stimulate CD4^+^ T cell were reported in postinfectious IBS mouse (61). Dendritic cells isolated from mesenteric lymph node of acetic acid and restraint stress-IBS rat and cocultured with splenic CD4^+^/CD8^+^ T cells showed an increase proliferation of T cells with a rise in secretion of IL-4 and IL-9 (62). Another study, using colorectal distension + restraint stress IBS rat, showed an increase in CD103-positive cells and proinflammatory cytokine IL-4 and IL-9 in colon. Mesenteric lymph node-dendritic cell, cocultured with CD4^+^ T cells and CD8^+^T cells, showed an increase IL-4 expression in CD4^+^ T cells and increase IL-9 expression in CD8^+^T cells. *In vitro* studies, using JAWSII cells, demonstrated that dendritic cells have the ability to produce and secrete CRF. Commensal bacterial strains can stimulate the production of CRF in dendritic cell, showing that CRF derived from dendritic cell may be involved in the pathogenesis of IBS (36, 37). JAWSII cells and mouse mesenteric lymph node-dendritic cells expressed CRFR1 and CRFR2 receptors (30, 38). CRF promotes inflammation in mature JAWSII by increasing the production of proinflammatory IL-6 and MIP-1α and decreasing anti-inflammatory IL-4 (30). CRF increases the capacity of mouse mesenteric lymph node-dendritic cells to stimulate T-cell proliferation, and treatment of mesenteric lymph node-dendritic cells with CRFR1 antagonist yielded a reduced capacity to stimulate T cells (38).

Mast cells are distributed in the mucosa and they have a major role in the transition from innate to adaptive immunity. Activated mast cells release a variety of preformed or newly synthesized inflammatory mediators (including proteases, histamine, prostaglandins, 5-HT, cytokines/chemokines, chymase, CRF, and tryptase) and various factors such as microenvironment, physiological, and psychological stress influenced its phenotype and the release of inflammatory mediators (29, 63). Mast cells and neurons in the GI tract express CRF and its receptors (31, 40, 42, 45, 49–52) and an electron micrograph from IBS patient colonic mucosa has shown membrane–membrane contacts between degranulating mast cell and a nerve fiber, proving the complex integrated interactions between neurons and immune cells *via* the CRF signaling in the pathophysiology of IBS. Colonic mast cell infiltration and mediator release modulating the intestinal nerve activity have been positively correlated with severity and frequency of abdominal pain in IBS patients (10). Some papers have reported elevated number of mast cell, whereas others have reported an unchanged number of mast cells (3). Investigators have shown the participation of TNF-α, a mucosal mast cell mediator, in tight junction dysregulation, and altered intestinal permeability (13, 64). In accordance with this finding, the elevated release of tryptase, histamine, and prostaglandin coexisting with increased paracellular permeability have been reported in colonic biopsies of IBS patients (4). Mast cells are immunocytochemically positive for CRH, and RT-PCR data have indicated presence of CRFmRNA in human cord blood-derived cultured mast cells. The presence of CRFR1 and CRFR2 in human and rat colonic mast cells are also supported by functional reports (13, 50). Chronic social stress in pigs resulted in reduced transepithelial electrical resistance in the ileum, and increased fluoresce flux in the ileum and colon. This stress also upregulated CRFmRNA in ileum and IL-10mRNA and mast cell chymase gene in both ileum and colon (65). In distal colon segments in Wistar–Kyoto rat, CRF induced a dose-dependent increase in short-circuit current (ion secretion), enhanced horseradish–peroxidase–flux and release of mast cell protease II. Non-selective CRFR1/R2 antagonist or mast cell stabilizer inhibited these epithelial responses. Mast cell-deficient rats show a reduced epithelial response to stress (59). Mast cell stabilizer alleviates barrier disruption-induced by water-avoidance stress and mucosal tissue exposed to CRF in colon, ileal villus epithelium, and follicle-associated epithelium of rat (56). Restraint stress or CRF administration followed by rectal distention enhanced the number of abdominal cramps and the colonic histamine content. CRF antagonist blocked the stress and CRF-induced enhancement of abdominal cramps (66).

T cells form part of the adaptive immune system and they are subdivided into different phenotypes including cytotoxic (CD8^+^), T helper (CD4^+^), memory (CD4^+^, CD8^+^, CD45RO), and regulatory (CD25^+^) based upon specific cell markers (29). The immune cells are mainly found in the intestinal mucosa (67). It has been reported that IBS patients have a greater amount of T cells, which are orienting to the GI tract. Furthermore, there is an increase in activated T cells expressing CD69 and HLA-DR residing in the colon IBS patient. Increased T-cell numbers in IBS patient’s biopsies of descending colon correlate with abnormal bowel movement (3). It has also been demonstrated that T lymphocytes have a major role in mediating changes in neuromuscular function following GI infection (68). Rise in T lymphocyte in *Campylobacter* enterocolitis patients is positively correlated with gut permeability. This description also matched changes observed in patients suffering from prolonged IBS symptoms after contracting acute bacterial enteritis (63). Convergent evidence indicates that human lymphocytes have the ability to secrete CRF, and CRF participates in immune system regulation by acting locally as a proinflammatory mediator (42). It has been reported that human T lymphocytes contain immunoreactive CRF (32) and express CRF gene. Stimulation with PHA/TPA leads to an increase in CRHmRNA levels, which decreased after 22 h (53).

B cells have a key role in the adaptive immunity as they have the ability to secrete a wide range of antibody, which protects the body against infections. Not only can B cells produce antibody but also act as antigen-presenting cells. IgA made by mucosal B cell is secreted to protect mucosal surfaces and the intestinal tissue against pathogens and food antigens. B cells derived from blood of IBS patients displayed augmented B-cell activation with increased cell surface expression of IgG, CD80, and CD86. UV-light-inactivated probiotic bacteria and LPS-exposed-B cells showed impaired ability to express costimulatory molecule CD80 (69). In IBS patients, immunoglobulin-producing B cells are involved in low-grade local GI inflammation (3). IBS patients have a lower number of IgA-B cells in the ascending colon and also a decreased number of IgA-B cells (vs. sigmoid colon) (70). Patients with IBS-D display a higher density of germline transcripts (GLTs) and activated B lymphocytes in the jejunal mucosa, which are predominantly distributed along the crypts in the lamina propria. A slight increase in IgA and IgM concentrations in the jejunal content was also reported in IBS-D patients (71). There is evidence proving that B lymphocytes have the ability to secrete CRF. In response to hyperthermia, hyperosmolarity and hypoxia stress, human T and B lymphocytes secrete CRF which is inhibited by corticosteroids (32, 33).

## Conclusion

This review summarizes the etiology of the onset of IBS referring to evidence that it may be triggered by diet/luminal microbiota, low-grade inflammation, stress, and genetic composition. Various animal models using chemical, mechanical stimulation, and physiological/psychological stress support that stress affects epithelial secretion, GI motility, inflammatory response, microbiota, abdominal muscle contraction, and response to pain. CRF and CRF receptors are distributed in immune cells, secretory cell, and tissues in the GI tract. Stress *via* local CRF system can activate cells in the GI tract and this can cause IBS phenotypes such as increase permeability, ion secretion, mucin secretion, visceral hypersensitivity, and release of proinflammatory cytokines. T cells and B cells have been shown to contribute to IBS progression. Further studies on mechanism network of central CRF system (circulation, sympathetic, parasympathetic neurons) and the local GI CRF system will provide new insights in understanding brain–gut axis.

## Author Contributions

MC and XC drafted the manuscript. XC and JD supervised the project and conceived of the student. JC contributed to discussions and revisions. ZM and YL contributed to figure, and table, and manuscript editing.

## Conflict of Interest Statement

The authors declare that the research was conducted in the absence of any commercial or financial relationships that could be construed as a potential conflict of interest.
